# Prediction of early recurrence of HCC after hepatectomy by contrast-enhanced ultrasound-based deep learning radiomics

**DOI:** 10.3389/fonc.2022.930458

**Published:** 2022-09-28

**Authors:** Hui Zhang, Fanding Huo

**Affiliations:** ^1^ Department of Ultrasound, Nanchong Central Hospital, The Second Clinical Medical College, North Sichuan Medical College (University), Nanchong, Sichuan, China; ^2^ Department of Medical Ultrasound, Sichuan Provincial People's Hospital, University of Electronic Science and Technology of China, Chengdu, China; ^3^ Chinese Academy of Sciences Sichuan Translational Medicine Research Hospital, Chengdu, China

**Keywords:** contrast-enhanced ultrasound, radiomics, AFP, hepatocellular carcinoma, early recurrence, deep learning

## Abstract

**Objective:**

This study aims to evaluate the predictive model based on deep learning (DL) and radiomics features from contrast-enhanced ultrasound (CEUS) to predict early recurrence (ER) in patients with hepatocellular carcinoma (HCC).

**Methods:**

One hundred seventy-two patients with HCC who underwent hepatectomy and followed up for at least 1 year were included in this retrospective study. The data were divided according to the 7:3 ratios of training and test data. The ResNet-50 architecture, CEUS-based radiomics, and the combined model were used to predict the early recurrence of HCC after hepatectomy. The receiver operating characteristic (ROC) curve and calibration curve were drawn to evaluate its diagnostic efficiency.

**Results:**

The CEUS-based radiomics ROCs of the “training set” and “test set” were 0.774 and 0.763, respectively. The DL model showed increased prognostic value, the ROCs of the “training set” and “test set” were 0.885 and 0.834, respectively. The combined model ROCs of the “training set” and “test set” were 0.943 and 0.882, respectively.

**Conclusion:**

The deep learning radiomics model integrating DL and radiomics features from CEUS was used to predict ER and achieve satisfactory performance. Its diagnostic efficiency is significantly better than that of the single model.

## Introduction

The most common primary liver cancer is hepatocellular carcinoma (HCC) (1). HCCs are the third most common cause of cancer-related death and ranked sixth in terms of incident cases (2). Owing to the wide prevalence of hepatitis B, China has high incidence and mortality rates. Its mortality rate is only second (3, 4) to lung cancer. Although technologies for the diagnosis and treatment of HCC are evolving, its 5-year recurrence rate is as high as 60%–80% (1, 4, 5). Hepatectomy is a common choice for treating HCC; the 5-year survival rate was <15% (6). Early recurrence (ER) was defined as the recurrence occurring after the first year during follow-up; it is considered to be an important factor affecting the survival rate of patients (7–9). In clinical practice, there is a need to assess the risk of ER in order to guide further monitoring and treatment (10).

Contrast-enhanced ultrasound (CEUS) has the advantages of high repeatability, non-invasiveness, no radiation, extremely low incidence of adverse reactions, and no hepatorenal toxicity, and can reflect the continuous dynamic perfusion of tumor in real time. It is often used to predict the pathological characteristics that are related to the risk of ER: size, microvascular invasion, and differentiation (11–14). In recent years, CEUS has been tried to predict the ER of HCC before operation (15, 16), but the effect is not satisfactory. Radiomics is an emerging research field aiming to use the full potential of medical images (17). It can be used for high-throughput extraction of quantitative features such as shape, gray, texture, and wavelet in medical images (18, 19). Although radiomics is widely used in the diagnosis and prognosis by computed tomography (CT) and magnetic resonance imaging (MRI) (20–22), deep learning has attracted extensive attention given its high performance in image recognition. In fact, it can effectively improve the diagnostic accuracy of medical image interpretation and the objectivity of diagnosis (23, 24). The combination of DL classification network and radiomics framework in the integrated system has become an emerging trend to achieve good performance in clinical tasks (25–27). However, there are no studies on CEUS-based deep learning radiomics analysis ER of HCC after hepatectomy. The purpose of this study was to explore the ability of CEUS-based DL to predict ER of HCC before operation.

## Methods

**Figure 1:**
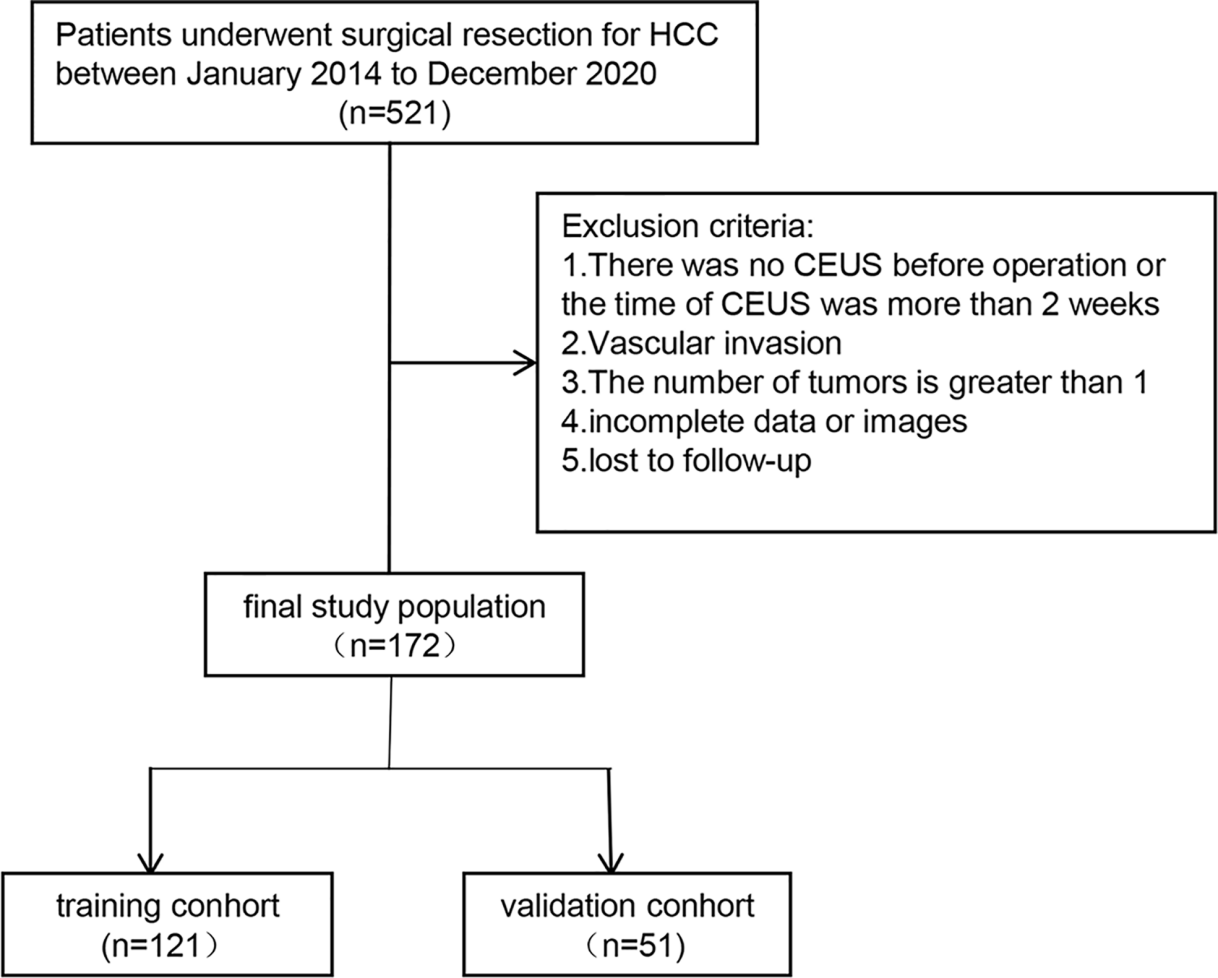
The flow chart is illustrated.

This retrospective study was approved by the research ethics committee. Each patient signed written informed consent before the examination. We collected data from 172 patients with HCC who underwent complete resection. All 172 patients with HCC underwent CEUS within 1 week before hepatectomy. Age, sex, history of hepatitis B or C, and AFP (<20µg/L,200-400µg/L) were included in the study. The dataset was assigned randomly to the training cohort or test cohort at a ratio of 7:3. The flow chart is illustrated in **Figure 1**.

### Follow-up

All patients were followed up, and abdominal US or CT scanning and AFP were performed every 3 months. A recurrence is considered when a recurrent lesion in or outside the liver is determined by imaging technology. The primary endpoint was tumor ER, which was defined as new intra- or extrahepatic tumors occurring within 1 year after surgery (9, 16). If recurrence occurred after the first year during follow-up, the endpoint of the study was considered to be ER. The definition of recurrence-free was 1-year period from the date of surgery to the date of first recurrence or metastasis.

### Ultrasonic examination

The US research was performed in Philips IU22 and EQIC7 systems and GE LOGIQ E9. All patients participating in this study fasted for a minimum of 8 h before the CEUS examination. The patients were asked to breath calmly. First, the upper abdomen was examined using grayscale US to locate the mass to be studied, and then, 2.4 ml of SonoVue was injected through the elbow vein by 5–10 ml of 0.9% saline. CEUS examination used low mechanical index (MI<0.1). The target lesion was continuously observed, and at least 3 min of digital movie clips were stored on the hard disk. According to the guidelines, it was divided into the arterial phases (AP) (0–30 s) and portal phases (PP) (30–120 s) (28). We selected three images for analysis: the tumor with US, the tumor in the AP on CEUS, and the tumor in the PP on CEUS.

### Extraction of CEUS radiomics features and construction of CEUS radiomics model

The maximum section image of the lesion was imported into Mazda 4.6 software. Two radiologists with more than 5 years of experience in CEUS depict a region of interest (ROI). The CEUS radiomics features were extracted from the ultrasonic image ROI of each patient by Mazda software. The corresponding texture parameters were histogram parameters (mean value, variance, kurtosis, skewness, percentile, etc.), grayscale symbiosis matrix (energy, contrast, autocorrelation, entropy, symbiosis sum, etc.), run test (length non-uniformity, long run weight, short run weight, and grayscale heterogeneity), autoregressive model, wavelet transform (wavelet transform system), number (energy), and absolute gradient (gradient mean, variance, skewness, and kurtosis). The inter- and intra-class correlation coefficients (ICCs) were employed to evaluate the consistency and repeatability of the results delineated. The ROI selections were performed by two readers. There was a good agreement of the feature extraction when the ICCs was >0.75. The ROI selections of the remaining images was performed by reader 1.

All cases in the training cohort were used to train the prediction model. All cases in the test cohort were used to evaluate the performance of the model independently. Finally, z-score was adopted to standardize the data. First, features with ICCs >0.75 were retained. Then, the U-test and the least absolute shrinkage and selection operator (LASSO) were used to filter the most useful features further. Finally, the CEUS radiomics model was developed using random forest. The selected radiomics features were linearly combined with their weighting coefficients to generate a radiomics score for each patient.

### Deep learning model

The proposed CNN retained the ResNet-50 network structure, which pre-trained on Imagene and adapted it to fit US data. Before model training, image processing operations such as rotation, horizontal or vertical flipping, random clipping, and random channel shifting were applied for data augmentation to generate more training images. These operations resemble the diversity observed in the real-world data and prevent overfitting. All the images were resized to 224 × 224 pixels and normalized.

As a small dataset was available, we adopted transfer learning and fine-tuning. We also performed feature extraction using pretrained models to take advantage of features learned from models trained on large datasets in the same domain. We set the learning rate as 1e−5 and applied the logistic optimizer to update the network parameters with batch size 24. The maximum iteration step was set to 1,000, and the learning rate decayed by one-half at 2,000 and 4,000 steps. The output prediction results of the ResNet-50 network were used as the classification results, and the cross-entropy of the prediction results and the labels was calculated as the loss function. This was done by instantiating a pretrained model and adding a feature extraction FC layer. The last 1,000 nodes of the FC layer were replaced with a specifically designed one FC layer with transfer learning initialized weights. Then, the parameters of the pretrained model were frozen, and only the classifier weights were updated during training. Deep learning model learned the high throughput image features, which could make full use of all embedded information in US images. Convolution operations were used to extract the features associated with each image, and the classifier was trained to determine the image class according to extracted features. By supervised classification, the label of input images was used to fine-tune the network and update the parameters and finally led to the most relevant features in the FC layer.

### Feature selection and development of three prediction models

**Figure 2:**
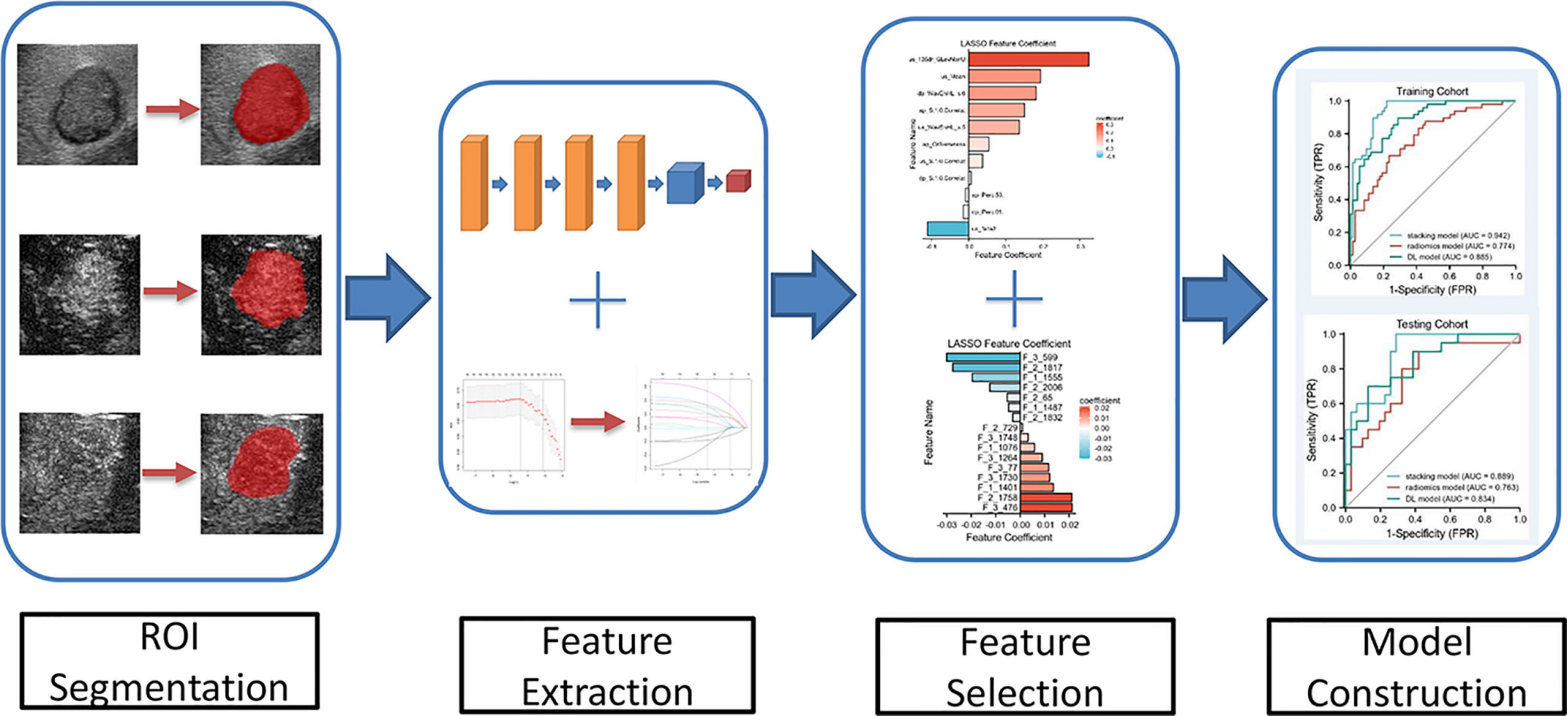
The flow chart of CEUS-based deep learning radiomics analysis for predicting ER of HCC is shown.

The ICC method, U-test, and LASSO with 10-fold cross-testing method were used to select characteristic parameters. We performed this process separately using previously extracted radiomics features and DL features to construct a radiomics model and the DL model, respectively. The RF was used to construct the radiomics model, and the support vector machine (SVM) was used to construct the deep learning model. Next, we performed a further post-fusion process using the radiomics model (RF algorithm) and the deep learning model (SVM algorithm) to make them jointly construct a combined prediction model. The architecture of the proposed CNN is shown in **Figure 2**.

### Statistical methods

The R3.3.2 (http://www.R-project.org) software and Python 3.7 (http://www.python.org) software were used to conduct data statistical modeling and analysis. Pearson chi-square test and Fisher’s exact test were also used. The p-values <0.05 were considered statistically significant. Statistically significant results obtained from univariate analysis will be submitted to multiple logistic regression. In this study, area under the curve (AUC), sensitivity, specificity, positive predictive value (PPV), and negative predictive value (NPV) of ResNet-50 model were used. Radiomics model and comprehensive model were calculated.

## Results

The clinicopathological features in the training set and testing test are shown in **Table 1**. A total of 172 patients and 68 cases had ER. Except for two cases of recurrent lesions in the lung and one case of bone metastasis, the rest of the 65 case recurred in the liver. Among 172 patients, according to the 7:3 segmentation data of training and test data, there were 121 patients in the training group, including 48 cases of recurrence within 1 year, and there were 73 cases without ER, with 104 men and 17 women, aged 48.3 ± 13.2 years. There were 51 cases in the training set, of which 20 cases recurred within 1 year and 31 cases had no ER; there were 43 men and 8 women, aged 52.9 ± 13.1 years.

**Table 1:** The clinicopathological features in the training and testing tests.

Characteristic	Training	Testing
**Number**	121	51
**Age**	48.3 ± 13.2	52.9 ± 13.1
**Sex**		
Male	104	43
Female	17	8
**Size(**cm**)**		
≤5	62	25
>5	59	26
**Hepatitis**		
Positive	115	46
Negative	6	5
**Cirrhosis**		
Positive	93	36
Negative	28	15
**AFP(**ng/ml**)**		
<20	39	16
20–400	29	11
>400	53	24
**ER**		
Positive	48	20
Negative	73	31

### Feature extraction and screening, and CEUS-based radiomics model development

**Figure 3:**
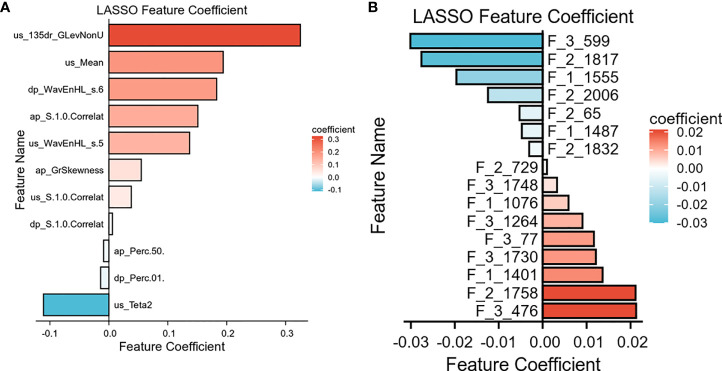
Characteristics selected the radiomics and DL model. **(A)** 11 features and its coefficients for the radiomics model. **(B)** 16 features and its coefficients for the DL model.

A total of 918 CEUS radiomics features were extracted from each patient’s ultrasound image ROIs using the Mazda software package. Mann–Whitney U-test (non-normal variables) was performed on the omics features, and 543 features were eliminated (p>0.05). A highly collinear relationship existed between omics features. Before modeling, Pearson or Spearman correlation analysis was conducted on the remaining 375 image features. If the correlation of the two variables was more than 0.6, the variable with an overall higher correlation was excluded, and 14 features were eliminated. After LASSO regression analysis, 11 meaningful radiomics features were finally obtained (**Figure 3A**). The CEUS radiomics score was created according to the following formula: CEUS radiomics score= −0.111× US_Teta2-0.014× DP_Perc.01.-.009× AP_Perc.50.+0.006×PP_S.1.0.Correlat0.038×US_S.1.0.Correlat+0.055×AP_GrSkewness0.137×US_WavEnHL_s.5+0.151×AP_S.1.0.Correlat0.183×PP_WavEnHL_s.6+0.194×US_Mean0.325×US_135dr_GlevNonU.

A total of 7,144 deep learning features were extracted from each patient’s ultrasound images (from three type of US images) by the ResNet-50 network. The same feature selection steps were performed to select the most significant features. Finally, 16 deep features were retained to construct the further model (**Figure 3B**). The combined model was constructed based on 11 radiomics features and 16 depth features.

### Diagnostic efficacy of three models in ER

**Figure 4:**
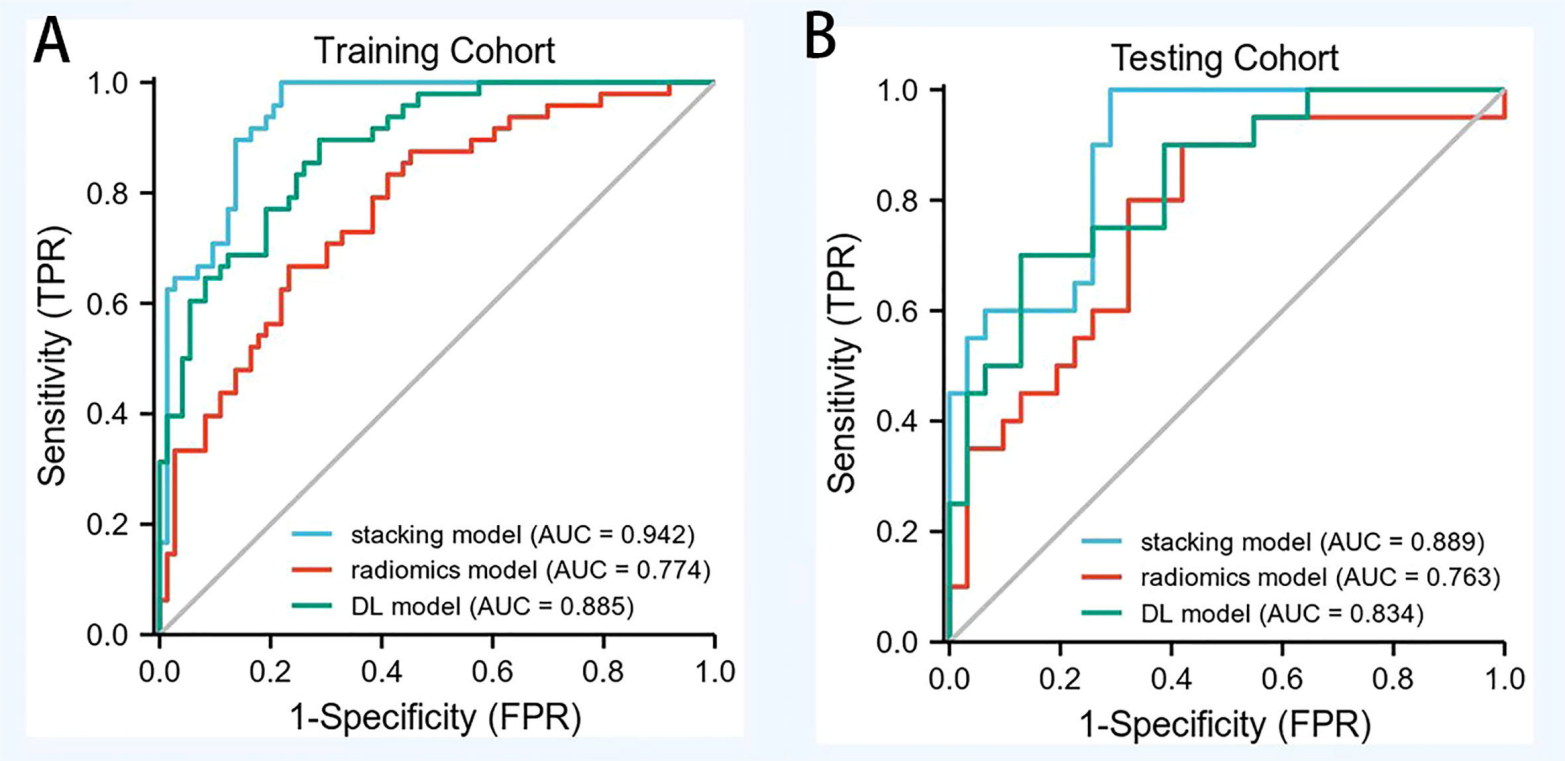
The ROC of radiomics model, DL model and combined model in training cohort and testing cohort. **(A)** AUC of combined models in training cohorts (AUC of 0.911) was significantly higher than that of the radiomics model (AUC of 0.740) and DL model (AUC of 0.887). **(B)** AUC of combined models in testing cohorts (AUC of 0.840) was significantly higher than that of the radiomics model (AUC of 0.780) and DL model (AUC of 0.813).

**Figure 5:**
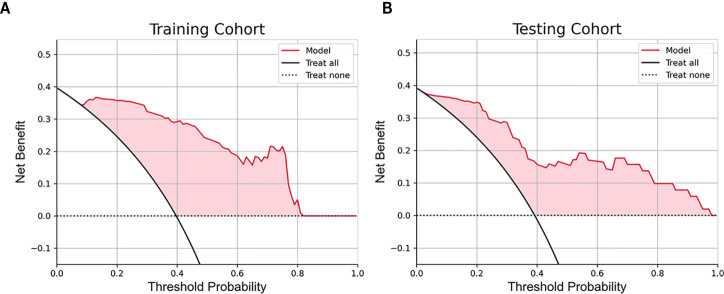
The decision curve analysis for the combined model. **(A)** The decision curve analysis in the training cohort. **(B)** The decision curve analysis in the testing cohort.

The ROC curve for predicting the ER of HCC by CEUS radiomics was drawn; the AUCs of the training and testing samples were 0.774 and 0.763 (**Figure 4**), respectively. The accuracy, sensitivity, and specificity of the CEUS-based radiomics score in predicting ER of HCC were 0.727, 0.667, and 0.767, respectively, in the training cohort and 0.686, 0.600, and 0.742, respectively, in the testing cohort. In the CEUS-based DL model, the AUC in the training and testing cohorts were 0.885 and 0.834, respectively. The accuracy, sensitivity, and specificity of the CEUS-based DL in predicting ER of HCC were 0.785, 0.896, and 0.712, respectively, in the training samples and 0.686, 0.800, and 0.613, respectively, in the testing samples; stacking model based on the radiomics model and the deep learning model, the AUCs in the training and testing cohorts were 0.942 and 0.889, respectively. The accuracy, sensitivity, and specificity of the CEUS-based DL radiomics model in predicting ER of HCC were 0.868, 1.000, and 0.781, respectively, in the training cohort and 0.784, 0.900, and 0.710, respectively, in the testing cohort (**Table 2**). The decision curve analysis showed that the combined model predicts a good overall net income of ER (**Figure 5**).

**Table 2:** Performance of training and testing sets in three models.

Cohort	Model	AUC (95%CI)	Accuracy	Sensitivity	Specificity
	Radiomic	0.774	0.727	0.667	0.767
**Train**	ResNet50	0.885	0.785	0.896	0.71
	Deep Learning Radiomics	0.942	0.868	1	0.78
	Radiomic	0.763	0.69	0.600	0.742
**Testing**	ResNet50	0.834	0.69	0.8	0.613
	Deep Learning Radiomics	0.889	0.784	0.9	0.667

## Discussion

Hepatectomy is the most effective treatment for HCC; ER within 1 year is now recognized as a critical determinant for a poor prognosis (29, 30). In this study, we evaluated the applicability of radiomics model based on CEUS and CEUS DL for the ER of HCC. A CUES-based DL radiomics using 11 features from radiomics and 16 features from DL analysis exhibited satisfactory performance; the predictive AUCs on ER of HCC of the training and testing samples were 0.942 and 0.889, respectively. Simultaneously, the combination of radiomics and DL to establish the model demonstrates a better effect than a single model. The CEUS DL is easy to use and can promote personalized risk stratification and further treatment decision-making in patients with HCC.

The ER of HCC may be due to micrometastasis and invasive tumor biological behavior (31). SonoVue is a blood pool contrast agent, which possibly reflects the microvessel composition of HCC. Previous studies have demonstrated that the CEUS characteristics can effectively predict the MVI and differentiation of HCC (13, 32, 33); these results suggest that the characteristics of CEUS are closely related to the ER. Qin’s research found that washout is a predictor of ER of HCC (16). The risk of ER increases with the flushing speed. Washout represents a more invasive vascular composition to a certain extent. Hai bin Tu also believes that Li-Rads can effectively predict the ER of HCC (15). However, the classification is subjective and depends on the experience of the radiologist. Radiomics transforms medical images into high-throughput features to quantitatively evaluate tumor phenotypes (18). This technology can extract quantitative image features using a computer algorithm to detect high-dimensional image features. Therefore, imageomics is objective compared with the conventional image interpretation of radiologists. The former can thoroughly analyze the whole tumor and obtain equivalent or more information than that obtained using conventional radiology (34). Imaging features reflect the texture information of the tumor and are important signs of intratumor heterogeneity. Intratumoral heterogeneity may result from genomic heterogeneity, associated with poor prognosis (32). Our study extracted the radiomics features of lesions from CEUS images. The radiomics score is based on 11 selected radiomics features, further indicating the tumor texture characteristics. Our results demonstrate that the CEUS-based radiomics score can effectively predict the ER of HCC. ResNet-50 has been used in feature extraction with high stability and performance for medical image classification (35). ResNet-50 can detect and classify lesions by setting an anchor and a bounding box without separating the foreground from the background. Moreover, it can extract features directly from the lesion area and then combine multiple features to classify lesions (36). Furthermore, integrating DL and radiomics algorithms can achieve higher diagnostic efficiency than a single method, and its benefits have been reflected in different clinical applications (26, 37). By integrating ResNet50 and radiomics models, we have improved our diagnostic ability, with an AUC of 0.889, which is higher than that of a single model.

Additionally, the combined model demonstrates satisfactory distinction, which provides an easy-to-use, visual, and personalized tool for ER prediction, assisting the doctors in the early prediction of the ER of HCC and performing corresponding measures, which is highly significant for the effective treatment of patients.

Our retrospective study has some limitations. First, radiomics features are based on the CEUS images in the two phases, and some information may have been omitted. Second is due to the small sample size, which may lead to overfitting and low repeatability of the prediction results. Third, as ultrasonic examination primarily depends on the experience of operators, the difference in operators may lead to the difference in graphic quality.

In conclusion, we developed a DL radiomics model based on CEUS to predict ER and achieve satisfactory performance. Its diagnostic efficiency is significantly better than that of the radiomics model. The DL radiomics model can be used by clinicians to judge the risk of ER and more effectively manage patients after surgery.

## Data availability statement

The original contributions presented in the study are included in the article/**Supplementary Material**. Further inquiries can be directed to the corresponding author.

## Ethics statement

This study was reviewed and approved by The Second Clinical Medical College, North Sichuan Medical College (University). The patients/participants provided their written informed consent to participate in this study.

## Author contributions

HZ and FH designed, coded, and tested the software tools and performed the analyses. HZ and FH provided the data and validated as expert the results. HZ and FH wrote the manuscript. All authors contributed to the article and approved the submitted version.

## Funding

Disclosure of grants of other funding Sichuan Provincial Administration of Traditional Chinese Medicine (2018QN33).

## Conflict of interest

The authors declare that the research was conducted in the absence of any commercial or financial relationships that could be construed as a potential conflict of interest.

## Publisher’s note

All claims expressed in this article are solely those of the authors and do not necessarily represent those of their affiliated organizations, or those of the publisher, the editors and the reviewers. Any product that may be evaluated in this article, or claim that may be made by its manufacturer, is not guaranteed or endorsed by the publisher.
