# Supplementary material for: Prediction of early recurrence of HCC after hepatectomy by contrast-enhanced ultrasound-based deep learning radiomics
Source: Front Oncol. 2022 Sep 28;12:930458. doi: 10.3389/fonc.2022.930458 (PMC9554932; doi:10.3389/fonc.2022.930458)
Supplement: Supplementary file 1 [file DataSheet_1.pdf]

Features Calculation

(1) Shape-based features

In this group of features, we included descriptors of the three-dimensional shape and size of the tumor region. Let in the following definitions V denote the volume and A the surface area of the volume of interest. We determined the following shape and size based features:

1. **Compactness 1**= $\frac{V}{\sqrt{\pi}A^{\frac{2}{3}}}$
2. **Compactness 2**= $36\pi\frac{V^2}{A^3}$
3. **Maximum 3d diameter**: The maximum three-dimensional tumor diameter is measured as the largest pairwise Euclidean distance, between voxels on the surface of the tumor volume.
4. **Spherical disproportion** = $\frac{A}{4\pi R^2}$
5. **Sphericity** = $\frac{\pi^{\frac{1}{3}}(6V)^{\frac{2}{3}}}{A}$
6. **Surface area**: The surface area is calculated by triangulation (i.e. dividing the surface into connected triangles) and is defined as:

   $$A = \sum_{\mathrm{i}=1}^{N}\frac{1}{2}\left|a_ib_i \times a_ic_i\right|$$

   Where N is the total number of triangles covering the surface and a, b and c are edge vectors of the triangles.
7. **Surface to volume ratio** = $\frac{A}{V}$
8. **Volume**: The volume (V) of the tumor is determined by counting the number of pixels in the tumor region and multiplying this value by the voxel size.

(2) First order statistical features

The following 17 statistical features were extracted.

Let **X** be the three dimensional image matrix with *N* voxels of the ROI and P be the first order histogram distribution with N_g discrete intensity levels.

1. **IntensityMax:** The maximum intensity value of **X.**
2. **IntensityMin:** The minimum intensity value of **X**.
3. **Median:** The median intensity value of **X**.
4. **IntensityStd:**

$$IntensityStd = \left(\frac{1}{L*W*H-1}\sum_{i=1}^{L}\sum_{j=1}^{W}\sum_{k=1}^{H}(X(\mathrm{i,j,k}) - IntensityAve)^2\right)^{1/2}$$

5. **Mean:**

$$\frac{1}{N}\sum_{i}^{N} X(i)$$

6. **Variance:**

$$\sqrt{\sum_{j=1}^{N_g} P(\mathrm{j}) * (\mathrm{j} - \sum_{i=1}^{N_g} P(\mathrm{i}) * \mathrm{i})^2}$$

7. **Skewness:**

$$\sqrt{\sum_{j=1}^{N_g} P(\mathrm{j}) * (\mathrm{j} - \sum_{i=1}^{N_g} P(\mathrm{i}) * \mathrm{i})^3}$$

8. **Kurtosis:**

$$\sqrt{\sum_{j=1}^{N_g} P(\mathrm{j}) * (\mathrm{j} - \sum_{i=1}^{N_g} P(\mathrm{i}) * \mathrm{i})^4}$$

9. **Range:**

   The range of intensity values of **X**.

10. **Mean absolute deviation:**

    The mean of the absolute deviations of all voxel intensities around the mean intensity value

11. **Energy:**

$$\sum_{i}^{N} X(i)^2$$

12. **Entropy:**

$$\sum_{i=1}^{N_g} P(i)\log_2 P(i)$$

13. Entropy_p:

$$\sum_{i=1}^{N_g} \frac{P(i)}{N}\log_2 \frac{P(i)}{N}$$

14. Root mean square:

$$\sqrt{\frac{\sum_i^N X(i)^2}{N}}$$

15. Uniformity:

$$\sum_{i=1}^{N_g} P(i)^2$$

16. **Uniformity_p:**

$$\sum_{i=1}^{N_g} (\frac{P(i)}{N})^2$$

17. Mass:

The sum intensity value of X.

(3) Textural features

Second order statistic texture features, and higher order statistic texture features were extracted. Forty-four second order statistic texture features could be calculated from the Gray Level Co-occurrence Matrix (GLCM). Forty-six high order statistic texture features were calculated from the Gray Level Size Zone Matrix (GLSZM), Gray Level Run Length Matrix (GLRLM), and Neighborhood Gray Tone Difference Matrix (NGTDM). All of the GLCM, GLSZM, GLRLM, and NGTDM based texture feature were calculated using a 2D analysis and then averaged for all slices within the three-dimensional tumor volume.

Gray-Level Co-Occurrence Matrix based features (GLCM)

GLCM based features were second-order statistical texture features, which are

defined as a matrix *M* (*i, j; δ, θ*) to indicate the relative frequency with intensity values of pixels (*i* and *j*) at the distance of *δ* in direction *θ*.

Let:

M(*i, j*) be the co-occurrence matrix for an arbitrary *δ* and *θ*, set *δ=1 and θ=0 and 45*

N_g be the number of discrete intensity levels in the images, set as 25,

μ be the mean of *M*(*i, j*),

$m_x(\mathrm{i}) = \sum_{j=1}^{N_g} M(\mathrm{i, j})$ be the marginal row probabilities,

$m_y(\mathrm{i}) = \sum_{i=1}^{N_g} M(\mathrm{i, j})$ be the marginal column probabilities, and u_y ,μ_x, be the mean of m_x .and m_y

$HX = -\sum_{i=1}^{N_g} m_x(i)\log(m_x(i),$

$HY = -\sum_{i=1}^{N_g} m_y(i)\log(m_y(i),$

$HXY = -\sum_{i=1}^{N_g}\sum_{j=1}^{N_g} m(i,j)\log(m(i,j)),$

$HXY1 = -\sum_{i=1}^{N_g}\sum_{j=1}^{N_g} m(i,j)\log(m_x(i)m_y(j)).$

$HXY2 = -\sum_{i=1}^{N_g}\sum_{j=1}^{N_g} m_x(i)m_y(j)\log(m_x(i)m_y(j)).$

1. **Energy:**

$$\sum_{i=1}^{N_g}\sum_{j=1}^{N_g}[\mathrm{M(i, j)}]^2$$

2. **Contrast:**

$$\sum_{i=1}^{N_g}\sum_{j=1}^{N_g}(\mathrm{i-j})^2 * M(\mathrm{i, j})$$

3. **Entropy:**

$$\sum_{i=1}^{N_g}\sum_{j=1}^{N_g} M(\mathrm{i, j}) * \log_2 M(\mathrm{i, j})$$

4. **Homogeneity 1:**

$$\sum_{i=1}^{N_g}\sum_{j=1}^{N_g}\frac{M(\mathrm{i},\mathrm{j})}{1+|\mathrm{i}-\mathrm{j}|}$$

5. **Homogeneity 2:**

$$\sum_{i=1}^{N_g}\sum_{j=1}^{N_g}\frac{M(\mathrm{i},\mathrm{j})}{1+|\mathrm{i}-\mathrm{j}|^2}$$

6. **Correlation:**

$$\frac{\sum_{i=1}^{N_g}\sum_{j=1}^{N_g} ijM(\mathrm{i},\mathrm{j})-\mu_i(\mathrm{i})\mu_j(\mathrm{j})}{\sigma_x(\mathrm{i})\sigma_y(\mathrm{j})}$$

7. **Variance:**

$$\sum_{i=1}^{N_g}\sum_{j=1}^{N_g}(\mathrm{i}-\mu)^2 M(\mathrm{i},\mathrm{j})$$

8. **Sum Average:**

$$\sum_{i=2}^{2N_g} iM_{x+y}(\mathrm{i})$$

9. **Sum Entropy:**

$$-\sum_{i=2}^{2N_g} M_{x+y}(\mathrm{i})\log_2\left[M_{x+y}(\mathrm{i})\right]$$

10. **Dissimilarity:**

$$\sum_{i=1}^{N_g}\sum_{j=1}^{N_g}|\mathrm{i}-\mathrm{j}|\,\mathrm{M}(\mathrm{i},\mathrm{j})$$

11. **Inverse Difference Moment:**

$$\sum_{i=1}^{N_g}\sum_{j=1}^{N_g}\frac{\mathrm{M}(\mathrm{i},\mathrm{j})}{1+(\frac{|\mathrm{i}-\mathrm{j}|^2}{N^2})}$$

12. **Autocorrelation:**

$$\sum_{i=1}^{N_g}\sum_{j=1}^{N_g} ijM(\mathrm{i},\mathrm{j})$$

13. **Cluster Prominence**

$$\sum_{i=1}^{N_g}\sum_{j=1}^{N_g}\left[i+j-u_x-u_y\right]^4 M(\mathrm{i,j})$$

14. Cluster Shade

$$\sum_{i=1}^{N_g}\sum_{j=1}^{N_g}\left[i+j-u_x-u_y\right]^3 M(\mathrm{i,j})$$

15. Cluster Tendency

$$\sum_{i=1}^{N_g}\sum_{j=1}^{N_g}\left[i+j-u_x-u_y\right]^2 M(\mathrm{i,j})$$

16. Difference Entropy

$$-\sum_{i=0}^{N_g-1} M_{x-y}(i)\log_2\left[M_{x-y}(i)\right]$$

17. Maximum Probability:

$$\max\left\{M(i,j)\right\}$$

18. Sum variance

$$\sum_{i=2}^{2N_g}(i-SE)^2 M_{x+y}(i)$$

19. Informational measure of correlation 1 (IMC1):

$$\frac{HXY-HXY1}{\max\left\{HX-HY\right\}}$$

20. Informational measure of correlation 2 (IMC2):

$$\sqrt{1-\mathrm{e}^{-2(HXY2-HXY)}}$$

21. Inverse Difference Moment Normalized (IDMN):

$$\sum_{i=1}^{N_g}\sum_{j=1}^{N_g}\frac{M(i,j)}{1+(\frac{|i-j|^2}{N^2})}$$

22. Inverse Difference Normalized (IDN):

$$\sum_{i=1}^{N_g}\sum_{j=1}^{N_g}\frac{M(i,j)}{1+(\frac{|i-j|}{N^2})}$$

Gray Level Run Length Matrix based features (GLRLM)

GLRLM based features were high-order statistical texture feature, which were defined as *P*(*i, j; θ*) to indicate the number of times j and gray level i appear consecutively in the direction *θ*.

Let:

P(*i, j; θ*) be the run-length matrix *P* for a direction *θ*, set *θ=0 and 45*

N_g be the number of discrete intensity values,

N_r be the number of different run lengths, and

N_p be the number of voxels in the ROI.

1. **Short Run Emphasis (SRE):**

$$\frac{\sum_{i=1}^{N_g}\sum_{j=1}^{N_r}[\frac{P(\mathrm{i},\mathrm{j};\theta)}{j^2}]}{\sum_{i=1}^{N_g}\sum_{j=1}^{N_r}P(\mathrm{i},\mathrm{j};\theta)}$$

2. **Long Run Emphasis (LRE):**

$$\frac{\sum_{i=1}^{N_g}\sum_{j=1}^{N_r}j^2P(\mathrm{i},\mathrm{j};\theta)}{\sum_{i=1}^{N_g}\sum_{j=1}^{N_r}P(\mathrm{i},\mathrm{j};\theta)}$$

3. **Gray-Level Nonuniformity (GLN):**

$$\frac{\sum_{i=1}^{N_g}[\sum_{j=1}^{N_r}P(\mathrm{i},\mathrm{j};\theta)]^2}{\sum_{i=1}^{N_g}\sum_{j=1}^{N_r}P(\mathrm{i},\mathrm{j};\theta)}$$

4. **Run-Length Nonuniformity (RLN):**

$$\frac{\sum_{j=1}^{N_r}[\sum_{i=1}^{N_g}P(\mathrm{i},\mathrm{j};\theta)]^2}{\sum_{i=1}^{N_g}\sum_{j=1}^{N_r}P(\mathrm{i},\mathrm{j};\theta)}$$

5. **Run Percentage (RP):**

$$\sum_{i=1}^{N_g}\sum_{j=1}^{N_r}\frac{P(\mathrm{i},\mathrm{j};\theta)}{N_p}$$

6. **Low Gray-Level Run Emphasis (LGRE):**

$$\frac{\sum_{i=1}^{N_g}\sum_{j=1}^{N_r}[\frac{P(\mathrm{i},\mathrm{j};\theta)}{i^2}]}{\sum_{i=1}^{N_g}\sum_{j=1}^{N_r}P(\mathrm{i},\mathrm{j};\theta)}$$

7. **High Gray-Level Run Emphasis (HGRE):**

$$\frac{\sum_{i=1}^{N_g}\sum_{j=1}^{N_r}i^2 P(\mathrm{i},\mathrm{j};\theta)}{\sum_{i=1}^{N_g}\sum_{j=1}^{N_r}P(\mathrm{i},\mathrm{j};\theta)}$$

8. **Short Run Low Gray-Level Emphasis (SRLGE):**

$$\frac{\sum_{i=1}^{N_g}\sum_{j=1}^{N_r}\frac{P(\mathrm{i},\mathrm{j};\theta)}{i^2 j^2}}{\sum_{i=1}^{N_g}\sum_{j=1}^{N_r}P(\mathrm{i},\mathrm{j};\theta)}$$

9. **Short Run High Gray-Level Emphasis (SRHGE):**

$$\frac{\sum_{i=1}^{N_g}\sum_{j=1}^{N_r}\frac{i^2 P(\mathrm{i},\mathrm{j};\theta)}{j^2}}{\sum_{i=1}^{N_g}\sum_{j=1}^{N_r}P(\mathrm{i},\mathrm{j};\theta)}$$

10. **Long Run Low Gray-Level Emphasis (LRLGE):**

$$\frac{\sum_{i=1}^{N_g}\sum_{j=1}^{N_r}\frac{j^2 P(\mathrm{i},\mathrm{j};\theta)}{i^2}}{\sum_{i=1}^{N_g}\sum_{j=1}^{N_r}P(\mathrm{i},\mathrm{j};\theta)}$$

11. **Long Run High Gray-Level Emphasis (LRHGE):**

$$\frac{\sum_{i=1}^{N_g}\sum_{j=1}^{N_r}i^2 j^2 P(\mathrm{i},\mathrm{j};\theta)}{\sum_{i=1}^{N_g}\sum_{j=1}^{N_r}P(\mathrm{i},\mathrm{j};\theta)}$$

12. **Mean:**

$$\frac{1}{2N_{\mathrm{g}}}\sum_{i=1}^{N_g}\sum_{j=1}^{N_g}[P(\mathrm{i},\mathrm{j})]^2$$

13. **Entropy:**

$$\sum_{i=1}^{N_g}\sum_{j=1}^{N_g} P(\mathrm{i},\mathrm{j}) * \log_2 P(\mathrm{i},\mathrm{j})$$

14. Energy:

$$\sum_{i=1}^{N_g}\sum_{j=1}^{N_g} [P(\mathrm{i},\mathrm{j})]^2$$

Gray Level Size Zone Matrix based features (GLSZM)

GLSZM based features were high-order statistical texture features, which were defined as *P*(*i, j*) to indicate the areas of size j and gray level i.

Let:

P(*i, j*) be the size zone of matrix *P*,

N_g be the number of discrete intensity values,

N_r be the number of different areas sizes,

N_p be the number of voxels in the ROI.

1. **Small Zone Emphasis (SZE):**

$$\frac{\sum_{i=1}^{N_g}\sum_{j=1}^{N_r} [\frac{P(\mathrm{i},\mathrm{j})}{j^2}]}{\sum_{i=1}^{N_g}\sum_{j=1}^{N_r} P(\mathrm{i},\mathrm{j})}$$

2. **Large Zone Emphasis (LZE):**

$$\frac{\sum_{i=1}^{N_g}\sum_{j=1}^{N_r} j^2 P(\mathrm{i},\mathrm{j})}{\sum_{i=1}^{N_g}\sum_{j=1}^{N_r} P(\mathrm{i},\mathrm{j})}$$

3. **Gray-Level Nonuniformity (GLN):**

$$\frac{\sum_{i=1}^{N_g} [\sum_{j=1}^{N_r} P(\mathrm{i},\mathrm{j})]^2}{\sum_{i=1}^{N_g}\sum_{j=1}^{N_r} P(\mathrm{i},\mathrm{j})}$$

4. **Zone-Size Nonuniformity (ZSN):**

$$\frac{\sum_{j=1}^{N_r}[\sum_{i=1}^{N_g} P(\mathrm{i,j})]^2}{\sum_{i=1}^{N_g}\sum_{j=1}^{N_r} P(\mathrm{i,j})}$$

5. **Zone Percentage (ZP):**

$$\sum_{i=1}^{N_g}\sum_{j=1}^{N_r} \frac{P(\mathrm{i,j})}{N_p}$$

6. **Low Gray-Level Zone Emphasis (LGZE):**

$$\frac{\sum_{i=1}^{N_g}\sum_{j=1}^{N_r}[\frac{P(\mathrm{i,j})}{i^2}]}{\sum_{i=1}^{N_g}\sum_{j=1}^{N_r} P(\mathrm{i,j})}$$

7. **High Gray-Level Zone Emphasis (HGZE):**

$$\frac{\sum_{i=1}^{N_g}\sum_{j=1}^{N_r} i^2 P(\mathrm{i,j})}{\sum_{i=1}^{N_g}\sum_{j=1}^{N_r} P(\mathrm{i,j})}$$

8. **Small Zone Low Gray-Level Emphasis (SZLGE):**

$$\frac{\sum_{i=1}^{N_g}\sum_{j=1}^{N_r} \frac{P(\mathrm{i,j})}{i^2 j^2}}{\sum_{i=1}^{N_g}\sum_{j=1}^{N_r} P(\mathrm{i,j})}$$

9. **Small Zone High Gray-Level Emphasis (SZHGE):**

$$\frac{\sum_{i=1}^{N_g}\sum_{j=1}^{N_r} \frac{i^2 P(\mathrm{i,j})}{j^2}}{\sum_{i=1}^{N_g}\sum_{j=1}^{N_r} P(\mathrm{i,j})}$$

10. **Large Zone Low Gray-Level Emphasis (LZLGE):**

$$\frac{\sum_{i=1}^{N_g}\sum_{j=1}^{N_r} \frac{j^2 P(\mathrm{i,j})}{i^2}}{\sum_{i=1}^{N_g}\sum_{j=1}^{N_r} P(\mathrm{i,j})}$$

11. **Large Zone High Gray-Level Emphasis (LZHGE):**

$$\frac{\sum_{i=1}^{N_g}\sum_{j=1}^{N_r} i^2 j^2 P(\mathrm{i,j})}{\sum_{i=1}^{N_g}\sum_{j=1}^{N_r} P(\mathrm{i,j})}$$

12. **Gray-Level Variance (GLV):**

$$\sum_{i=1}^{N_g}\sum_{j=1}^{N_r}\{\mathrm{iP(i,j)} - \frac{\sum_{i=1}^{N_g} i[\sum_{j=1}^{N_r} P(\mathrm{i,j})]}{N_g N_r}\}$$

13. **Zone-Size Variance (ZSV):**

$$\sum_{i=1}^{N_g}\sum_{j=1}^{N_r}\{\mathrm{jP(i,j)} - \frac{\sum_{i=1}^{N_g} i[\sum_{j=1}^{N_r} P(\mathrm{i,j})]}{N_g N_r}\}$$

Neighborhood Gray Tone Difference Matrix based features (NGTDM)

NGTDM based features were high-order statistical texture features, which were defined as *S(i)* to indicate the sum of the absolute value between gray intensity level i and it's neighbors' average intensity.

Let:

S(i) be the sum of absolute value between gray intensity level i and its neighbors' average intensity,

C(i) be the number of voxels with the gray intensity level I,

N_g be the number of discrete intensity values.

1. **Coarseness:**

$$\frac{1}{\varepsilon + \sum_{i=1}^{N_g} \frac{C(\mathrm{i})\mathrm{S(i)}}{\sum_{i=1}^{N_g} C(\mathrm{i})}}$$

2. **Contrast:**

$$\frac{\sum_{i=1}^{N_g}\sum_{j=1}^{N_g} C(\mathrm{i})\mathrm{C(j)(i-j)}^2}{(\sum_{i=1}^{N_g} C(\mathrm{i}))^2} * \sum_{i=1}^{N_g} S(\mathrm{i}) * \frac{1}{N_g(\mathrm{N}_g - 1)\sum_{i=1}^{N_g} C(\mathrm{i})}$$

3. **Busyness:**

$$\frac{\sum_{i=1}^{N_g} C(\mathrm{i})\mathrm{S}(\mathrm{i})}{\sum_{i=1}^{N_g}\sum_{j=1}^{N_g}(\mathrm{iC}(\mathrm{i})-\mathrm{jC}(\mathrm{j}))}, \quad C(\mathrm{i})\neq 0, \mathrm{C}(\mathrm{j})\neq 0$$

4. **Complexity:**

$$\frac{1}{(\sum_{i=1}^{N_g} C(\mathrm{i}))^2} * \sum_{i=1}^{N_g}\sum_{j=1}^{N_g}\frac{|\mathrm{i}-\mathrm{j}|(\mathrm{C}(\mathrm{i})\mathrm{S}(\mathrm{i})+\mathrm{C}(\mathrm{j})\mathrm{S}(\mathrm{j}))}{(\mathrm{C}(\mathrm{i})+\mathrm{C}(\mathrm{j}))}, \quad C(\mathrm{i})\neq 0, \mathrm{C}(\mathrm{j})\neq 0$$

5. **Strength:**

$$\frac{\sum_{i=1}^{N_g}\sum_{j=1}^{N_g}(\mathrm{C}(\mathrm{i})-\mathrm{C}(\mathrm{j}))*(\mathrm{i}-\mathrm{j})^2}{\sum_{i=1}^{N_g} C(\mathrm{i}) * \sum_{i=1}^{N_g} S(\mathrm{i})}, \quad C(\mathrm{i})\neq 0, \mathrm{C}(\mathrm{j})\neq 0$$

(4) Wavelet features: first order statistical and texture features of a wavelet filtered image.

Derived wavelet features were extracted from each image, with the Gaussian filter and a wavelet-based filter. These features were computed on the filtered images. The original image was filtered by specific filters we set. For each image, the first order statistical and texture features were computed again and the new features were wavelet-based features.

(5) Deep learning features

Deep learning features were extracted from the FC layer of the transfer learning model (ResNet-50) from input image. The three ResNet-50 based model shared the pre-training parameters, which accepted different kinds of US images as image inputs, respectively. The final convolutional layer outputs of the parallel network were concatenated and fused by the FC layers. Deep learning features were extracted from layers the same as one modal model. In particular, as our task was a classification task, the last FC layer was replaced with our specifically designed FC layers with Xavier initialized weights. Then 2024 features could be extracted from each image for each patient.
